# Views and experiences of discharged COVID-19 patients in Kano, Nigeria: a qualitative study

**DOI:** 10.11604/pamj.supp.2020.37.1.26609

**Published:** 2020-11-17

**Authors:** Naziru Bashir Mukhtar, Auwal Abdullahi, Muhammad Aliyu Abba, Jibril Mohammed

**Affiliations:** 1 Department of Physiotherapy, Faculty of Allied Health Sciences, Bayero University Kano, Kano, Nigeria,; 2 Department of Rehabilitation Sciences, Ghent University, Ghent, Belgium,; 3 Department of Rehabilitation Sciences and Physiotherapy, University of Antwerp, Movant, Wilrijk, Belgium,; 4 Department of Physiotherapy, College of Medicine, University of Ibadan, Ibadan, Nigeria

**Keywords:** COVID-19, patients, qualitative research, hospitalisation

## Abstract

**Introduction:**

COVID-19 has spread globally, thereby contributing to substantial hospitalisation rates and morbidity. However, little or no information is available on the experiences of patients with COVID-19 in an African-setting. The study aimed to explore the experiences of patients with COVID-19.

**Methods:**

semi-structured interviews were conducted via telephone with eleven individuals who were managed and discharged due to COVID-19. A descriptive phenomenological approach to qualitative research was employed and participants were mainly asked about their experiences before, during and after hospitalisation for COVID-19. Data were analysed using thematic analysis.

**Results:**

patients' viewpoints were suggestive of community and secondary transmission of COIVD-19 in the study area. A few participants experienced severe symptoms. Most participants tend to resign their condition to fate; while some displayed unfounded conspiracy theories. Nevertheless, precautionary measures to prevent infection were largely observed. COVID-19 also negatively affected activities of daily living of the participants. Furthermore, the participants were generally satisfied with quality of care provided. However, areas of patients' education, isolation centre set-up and caregiver-patient interaction needed further improvements. Lastly, experience of fear and stigma during post-hospitalisation were common.

**Conclusion:**

COVID-19 impacted negatively on the lives of the studied population. However, their experience during hospitalisation was generally positive.

## Introduction

Coronavirus disease (COVID-19), also known as severe acute respiratory syndrome coronavirus-2 (SARS-CoV-2) refers to a novel RNA coronavirus that emerged in late 2019 in Wuhan, China [1,2]. Ever since, COVID-19 has become a global public health threat, endangering the health and well-being of all people, especially those with existing comorbid conditions [3]. The disease has spread to most countries of the world, affecting millions of people and causing over 1.2 million deaths [4]. The most affected regions of the world include the Americas, Europe, Southeast Asia and the Mediterranean [4,5]. In Africa, the number of COVID-19 cases is comparatively lower than other regions; however, the trend of the disease is on a gradual increase in many countries [6]. According to the Africa Center for Disease Control and Prevention, there were over 1.6 million confirmed positive cases of COVID-19 recorded in Africa as of mid-October, 2020, out of which over 39,000 individuals had died; while some 1.3 million patients recovered from the disease [7]. In Nigeria, over 60,000 positive cases of COVID-19 have been diagnosed as of 14^th^ of October 2020, out of which, about 1,100 died due to the disease [8].

Nevertheless, Nigeria is rated as a vulnerable country to the disease due to its large population (potentially 200 million citizens) and weak health systems [9]. Moreover, the testing capacity and testing rates in Nigeria has been considered to be inadequate [10]. Thus, Nigeria is being described to have a moderate capacity to control outbreaks like COVID-19 [11]. The capacity to provide sufficient bed space and associated clinical care to support those who could need isolation and quarantine following local cycles of transmission of COVID-19 is also a subject of concern [9]. As of mid-August, 2020; Lagos State was the epicentre of the disease in Nigeria with over 16,000 positive cases out of which over 200 individuals died. This is followed by Abuja (the Federal Capital Territory [FCT]) that recorded about 4,700 cases and 45 deaths. Kano State was placed on the 7^th^ position (out of 37 states including the FCT) in Nigeria with over 1,700 total positive cases and 54 deaths [8].

Patients with COVID-19 often present with variable clinical features across various systems of the body including respiratory and digestive tract [12,13]. These symptoms may include mild self-limited disease to severe pneumonia, acute respiratory distress syndrome, septic shock, and systemic multiple organ failure syndrome. Consequently, some of the infected patients may require intensive care during isolation [2]. A comprehensive treatment package may be necessary to improve the experience of patients during COVID-19 hospitalization. For instance, provision of physical activity schedules and improving the aerobic capacity post COVID-19 have been utilized in many developed countries during the management of these patients [14link>]. Furthermore, patients managed on account of COVID-19 could provide relevant information that can be utilized to improve management and care, especially in resource limited settings where international guidelines may not be practicable. Therefore, our study aimed to explore the views and experiences of discharged patients who were managed on account of COVID-19 in Kano, Nigeria through semi-structured in-depth interviews. We envisage that, findings from this study will inform policy on how to improve current interventions in a patient-centered manner. The outcome of this study may also highlight valuable information on the experiences of COVID-19 patients from an African perspective.

## Methods

This qualitative study was reported in line with the consolidated criteria for reporting qualitative studies (COREQ) checklist [17]. A phenomenological approach to qualitative research, which summarizes individuals’ experiences and how they experienced them was used in this study. Phenomenology essentially focuses on the commonality of a lived experience or the life world of a particular group with a goal of creating meaning and achieving a sense of understanding [18,19]. Specifically, a descriptive phenomenology method was chosen in this study because it disallows the linking of prior knowledge, while calls for detailed description of lived experience without ascribing meaning is emphasized [20]. Descriptive phenomenology is also based on the meaning of the individual´s experiences that comprises of perception, thought, memory, imagination, and emotion all of which are termed as 'intentionality' [21]. Thus, the primary focus of this study was on patients´ experience and perspective about COVID-19 and its treatment.

**Participants:** we recruited Hausa-speaking patients that were admitted and discharged on account of COVID-19 from the major designated COVID-19 treatment centre in Kano, Nigeria. The telephone contacts of patients were obtained after seeking permission from the relevant authority in the treatment centre. Additional approvals were also obtained from the taskforce on COVID-19 in Kano State. The COVID-19 index case in Kano was admitted less than 3 months from the time of the interview (June, 2020), implying that all discharged patients were less than 3 months post-discharge. Therefore, all adult patients that were discharged from the treatment centre were qualified for inclusion into our study. Purposive sampling with emphasis on intensity sampling method was employed in recruiting the participants of this study [22]. Theoretical saturation was used to stop data collection from the patients [23].

**Interviewers:** to minimize interviewer dominance, interviews were conducted by two (male) researchers. However, the same device (recorder) was used to make the phone calls to the respective participants for the interview.

**Interview method:** prior to the interview-proper, the interviewers conducted a pilot interview to optimize and familiarize themselves with the questions and the themes. Interviews were repeated where necessary. Structured questions for the interview were developed by all authors through an online meeting via Zoom until the contents were considered adequate and were unanimously accepted. The interview question guides and demographics are presented in Table 1 and Table 2, respectively. The interviews were administered in Hausa language, and they were recorded using the same smart phone. However, no further interviews were conducted as soon as theoretical saturation was attained. Each interview lasted less than 18 minutes, which is similar to earlier reports for short telephone interviews [24]. Following the interviews, the responses of the participants were transcribed. Thereafter, forward and backward translation method was used in translating the transcribed interviews to English from Hausa [25]. The translation panel (the researchers) ensured that participants' style and meanings were preserved.

**Table 1:** interview guide

Question 1	How did you get infected with COVID-19 infection?
Question 2	What were your beliefs about COVID-19 before you got infected?
Question 3	What precautions did you take before you got infected?
Question 4	Tell us about the status of your health while on admission?
Question 5	What parts of your body or functions were affected by the disease?
Question 6	Can you describe your routine treatment and activities while on admission?
Question 7	Do you know the respective professions of the health workers that treated you while on admission?
Question 8	How can you describe your experience with the treatment as a whole?
Question 9	What areas do you think require improvement
Question 10	How does the infection affect your day-to-day activities presently?
Question 11	The essence of this study is to report on your experience with COVID-19, tell us anything you think we may have missed in our previous questions?

**Table 2:** demographic characteristics of the participants

Variables		Parameters
**Gender**	Male, N (%)	10(90.91)
	Female, N (%)	1(9.09)
**Age (years)**	Mean (SD)	44.64±15.15
	Range	21-70
**Marital status**	Single (%)	1(9.09)
	Married (%)	10(90.91)
**Duration of admission (days)**	Mean (SD)	19.91±6.59
	Range	12-31
**Co-morbidities**	Present N (%)	1(9.09)
	Absent N (%)	10(90.91)

**Data cleaning and analysis method:** the data collected was transcribed by an independent research assistant, who holds a Bachelor's degree in Library Science and has experience in interview transcription. Transcripts were then returned to the participants for comments or corrections. Thereafter, each transcript was reviewed and coded by two of the authors independently who then met afterwards to harmonize their results [26]. For the analysis, there are several approaches that are used when the aim is to describe the meaning of an experience through emergent themes within the different schools of phenomenology [21]. Since this study was based on Husserl´s descriptive phenomenology, the van Kaam's method, which require that inter subjectivity be confirmed through expert judges was used as the method of analysis [21,27,28]. Similarly, descriptive statistics was used to analyse the participants' personal and clinical information.

**Ethical clearance:** ethical approval was sought and obtained from the Human Research Ethics Committee, Ministry of Health, Kano State, Nigeria (MOH/Off/797/T.I/2020). The ethical approval was granted for the purpose of interviewing both discharged COVID-19 patients and healthcare providers of COVID-19 patients. Furthermore, permissions from the Kano State COVID-19 Taskforce and treatment centre were also obtained before undertaking this work.

## Results

**The participants:** a total of 11 patients were included in the study. Majority of the participants were males (90.9%) as only one participant was a female. The average age of the participants was 44.6 years (ranging between 21 to 70 years). The average duration of admission was about 3 weeks (19.9 days). Finally, majority (90.9%) of the participants were married, and a similar percentage had no comorbidities prior to hospitalization (Table 2). Table 3 provides the information on the participants commonalities discovered from the one-on-one interviews.

**Table 3:** commonalities of background information

Commonalities	Participants
Identified as discharged COVID-19 patient	All participants
Identified as male	All participants except AS2
Identified as female	AS2
Identified marital status as married	All participants except UAS
Identified as single	UAS
Identified with absence of comorbidities	All participants except AS2

**Thematic analyses:** this section provides a narrative of the themes generated from the analysis of the data to answer the research questions. Only participants´ responses that were in line with the interview questions were quoted. For clarity purpose, themes were categorized under the respective interview questions. The interview questions and the respective themes/quotes are presented in Table 4.

**Table 4:** thematic categories

Questions	Themes
How did you get infected with COVID-19 infection?	Community transmission
	Secondary transmission
What were your beliefs about COVID-19 before you got infected?	Conspiracy theories
	Trust
What precautions did you take before you got infected?	Social distancing
	Face-masking
	Hand hygiene
	Self-isolation
Tell us about the status of your health while on admission?	Symptomatic
	Asymptomatic
What parts of your body or functions were affected by the disease?	Activities of daily living restrictions
Can you describe your routine treatment and activities while on admission?	Environmental and patient hygiene
	Care and supervision
	Responsiveness to patient issues
	Conventional care
Do you know the respective professions of the health workers that treated you while on admission?	Recognition and identification
What areas do you think require improvement?	Care improvement
	Care providers welfare
	Patient centered care and education
	Patients-caregiver interaction
	Symptom based isolation
How does the infection affect your day-to-day activities presently?	Activities of daily living
	Felt-stigma, fear and anxiety

**Community and secondary transmission:** participants reported that they developed COVID-19 symptoms despite not having any history of travel or contact with a confirmed case. Some participants reported testing positive for COVID-19 after visiting the hospital for a different complaint. AS: “*I did not have any prior contact with a known or suspected case*.” AM: “*I had no previous contact with any known COVID-19 patient*.” AS2:“*I then started having fever and cough. In the hospital, I was tested for COVID-19 and the result was positive*.” A few other participants were able to attribute their positive result to either recent travel, following a condolence visit or after having contact with a person that later turned out to be a confirmed case. MK: “*I received a call from my friend that was with me at the condolence that he tested positive for COVID-19 because the first index case of Kano city was also at the same condolence visit. My sample was taken and the result came out positive*.” KA: “*My brother was tested positive for COVID-19 and I had very close contact with him. He then called us for testing and my result was positive*.”

**Beliefs and precautionary measures against COVID-19:** the participants seem to be divided in terms of beliefs about COVID-19. Some participants have the notion that the disease is deliberately created in the laboratory; while others attribute it only to rich folks who have the means to travel to developed countries. AS: “*I believe it is created*.” MK: “*My thought is that COVID-19 is not in Nigeria because in countries like China, America and the rest, they show the isolation centers and the patients, but in Nigeria we only hear about the number of cases increasing*.” Some other participants seem to have some knowledge, primarily from media sources, and therefore believed that the virus truly exists. However, a few participants also attributed the outbreak of COVID-19 to destiny. AM: “*I don´t argue about it. There were previous diseases like AIDs and so on. We were told they exist and we later see them*.” NIF: “*the information I read about COVID-19, I believe it exists and I have no doubt about it*.” MS: “*I am a Muslim, I believe in destiny. The way I see things happening worldwide, I know this disease is real*.” Nevertheless, there appears to be a general consensus in terms of good precautionary measures that each participant took against COVID-19. Measures taken include social distancing, avoiding crowds and the use of hand sanitizers and face masks. KA: “*For almost 2 months, I don´t go to mosque to join congregational prayers*” US: “*I don´t meet people except with a face mask till today*.” NIF: “I *always have hand sanitizer in my car and I use it*.”

**Experiences of patients during COVID-19 hospitalization:** some participants described their stay during hospitalization as a difficult time due to COVID-19 symptoms, especially among those with underlying disease conditions. These symptoms typically include dyspnoea (breathlessness) of different intensities and sometimes headache, fever, cold, body weakness, anosmia and pain. AS2: “*I had swellings and difficulty in breathing. But I have underlying heart disease*.” UAS: “*I was having a severe flu like symptoms initially with neck pain, but gradually I got better before I got discharged*.” NIF: “*During the early days in the isolation center, I had respiratory difficulty and I find it difficult to walk*.” MK: “*I was suffering from headache, fever, cold, body weakness, respiratory difficulty, cough and whatever you put in my mouth I will not perceive the smell/aroma*.” Nevertheless, some participants reported that they had no symptoms (asymptomatic) throughout their stay in hospitalization as a result of being infected with COVID-19. MS: “*I never had any symptom”* MJ: “*I did not reach any symptom level*.” The findings of this study also indicated that majority of the participants reported that they experienced restrictions in their routine activities of daily living. These activities include engaging in acts of worship, doing household chores, working and visiting friends and family. AS: “*if I wash, my breathing becomes a little difficult*.” AM: “after *the COVID-19, I find it difficult to recite the Qur´an*.” KA: “*I was also coughing which limits my ability to answer phone calls*.”

The study revealed that the participants were impressed with the environmental hygiene of the treatment centre and quality of care. AS: “*The center we were admitted was always clean*.” AS2:“*We received excellent treatment*.” UAS: “*We get our food and medications on time*.” The participants admitted that any problem that they had was quickly resolved by the concerned authorities. Some participants did not have problems with recognising their caregivers´ professions. NIF: “*when those problems were raised, they were solved very quickly and efficiently*.” UAS: “*Yes, we recognize the health care members. We also see names of staff members on their uniform*.” Another major finding of this study was that the participants reported that infrastructure, equipment, medications and gadgets that were either put in place or used were of high standard quality and good model. MK: “*They gave us medications that are special, of high quality and are expensive*.” MM: “*The medical equipment and gadgets I see at the isolation center are world standard*.”

**Suggestions for improvement of care and post-hospitalization experience:** the patients reported the need to improve in areas of patient's education, caregiver and patient interaction, and the need for government to improve on the welfare package of caregivers (health workers). The patients also called for the need to have separate isolation centres for symptomatic and asymptomatic COVID-19 patients. AM: “*There is need for improvement of the shower rooms and supply of soaps and body creams/lotions*.” NIF: “*government should be paying the healthcare providers their allowances/salaries as at and when due*.” UAS: “*Patients should receive adequate education about the symptoms, presentations and stages of the COVID-19 disease*.” MM: “*Patients that have symptoms and those without symptoms should have separate isolation centers*.” The participants reported that they faced difficulties in terms of their businesses and work following their discharge from the isolation centers. They also experience occasional fear when they remember their stay during hospitalization, and possible stigma, which makes it difficult for them to interact with people even after discharge. AM: “*It has completely crippled my business*.” MK: “*I am afraid of going to people and interacting with them after discharge from the isolation center*.” MS: “because *some patients that were with us in the isolation center have died, that fear is still in me*.”

## Discussion

This study was aimed at exploring the experiences of patients before, during and after hospitalization on account of COVID-19. The major outcomes of this study indicated that both community and secondary transmission are the major modes of transmission of COVID-19 among the participants. Some participants exhibited unfounded conspiracy theories regarding COVID-19; however, they reported taking recommended precautionary measures against the disease prior to being infected. The patients also recorded reduced limitation in their routine activities of daily living. However, the quality of care was reported to be optimal and described to be excellent by some participants. Finally, participants of the study reported that there was a need for improvement in areas of care-giver/patient interaction and welfare of the healthcare providers in the isolation centre. The findings of this study have added to the literature on the importance of patients' perspective in healthcare delivery [29]. This is because, unlike the result of the present study, previously, it was reported that, patients usually perceive healthcare providers to be hostile in developing countries like Nigeria [30]. In addition, the patients in the present study also sought for improved welfare for the attending health workers. We consider this result to be important because healthcare providers have continuously agitated for improved welfare package [31]. Therefore, bringing this information to the fore may be useful to relevant authorities seeking to enhance the quality of healthcare delivery in similar low resourced settings especially during this pandemic, a time when healthcare workers are afraid of risking their lives.

Similarly, both community and secondary transmission of COVID-19 were reported by the study participants. This could be explained by the high rates of conspiracy theories around COVID-19 in the study area and earlier forecast by previous researchers [32]. Moreover, a significant proportion of individuals still have the notion that COVID-19 is a hoax, or does not affect Africans like other populations [33]. Therefore, there is a need for further and continuous enlightenment of the populace using different means (modern and traditional) about scientific bases of diseases such as COVID-19. Furthermore, in general, the participants were impressed with the quality of care administered to them in the isolation centre. This is likely due to the serious nature of COVID-19 on several aspects of human life, which has led to an unprecedented galvanisation of global response to combat it [34,35]. Hence, the overall efforts in combating the disease by the federal and state governments in Nigeria may have led to the substantial improvement in quality of care reported in this study [36]. It is noteworthy that, many of the isolation centres are furnished with brand new and state of the art medical equipment. Moreover, a number of philanthropists continue to provide both financial and material support during the heat of the COIVD-19 pandemic [37]. Therefore, this can serve as a model to combat future epidemics or pandemics where all hands (the government, the business community, religious bodies and the general public) should be on deck.

Nevertheless, the use of other complementary interventions like rehabilitation (physiotherapy) that could help enhance physical activity and boost patients´ immunity was not reported by the participants. Physical activity has been reported to be of immense importance among COVID-19 patients [38]. Rehabilitation has been recommended for COVID-19 patients in both acute settings [39] and at post discharge [40], because of its beneficial effects on patients health outcomes and experiences. Furthermore, Abdullahi *et al*. [41] recently called for the need to adequately engage African physiotherapists in the management of COVID-19. Therefore, we recommend the use of more therapeutic options in a multidisciplinary manner for patients in similar settings in order to optimise patients' outcomes. The study has a number of areas of strengths. These include the exploration of viewpoints of patients within 3 months of discharge from hospitalization. This is important for capturing accurate and reliable information. In addition, all interviews were conducted by the same researchers, in order to increase the consistency of the data collection. Furthermore, coding was performed by the same researchers working independently in order to free the data from individual bias. Similarly, the participants had a lot of commonalities. Majority of them were males, married, and had no comorbidities thereby allowing our results to be viewed in a particular perspective. Also, the wide range in the age of the participants is important to capture the generational viewpoints. In contrast, the main limitation of this study is that only participants from one isolation centre participated in the study. However, this is unlikely to have any adverse impact on our study findings because the isolation centre that was used is one of the two major isolation centres in Kano, Nigeria. Moreover, we ensured that saturation of information was reached before stopping further interviews.

## Conclusion

In general, COVID-19 patients have diverse experiences largely influenced by their clinical presentations during hospitalisation. However, they appear to have a consensus among them that the level of care and health infrastructure was positive. Nevertheless, few areas bordering around care giver/patient interaction, welfare of health workers may warrant further improvement.

### What is known about this topic

- COVID-19 patients present with varied level of symptoms or none at all;
- In many settings in Africa, patients with COVID-19 are being managed successfully during hospitalization;
- COVID-19 affects many aspects of peoples' life including activities of daily living, mental health and work.

### What this study adds

- That the lived experiences of African patients before, during and after hospitalization for COVID-19 is generally positive;
- Patients with COVID-19 in the studied population perceive that the welfare of their healthcare providers is inadequate;
- Some individuals treated for COVID-19 experience socioeconomic losses as well as post-hospitalization stigma and fear.
